# Health-Related and Psychosocial Factors Associated with Prostate Cancer Stage at Diagnosis among Males Participating in Alberta's Tomorrow Project

**DOI:** 10.1155/2023/4426167

**Published:** 2023-11-10

**Authors:** Michelle L. Aktary, Brittany Shewchuk, Qinggang Wang, Eric Hyndman, Lorraine Shack, Paula J. Robson, Karen A. Kopciuk

**Affiliations:** ^1^Faculty of Kinesiology, University of Calgary, 2500 University Drive NW, T2N 1N4, Calgary, Alberta, Canada; ^2^Cancer Epidemiology and Prevention Research, Cancer Care Alberta, Alberta Health Services, T2S 3C3, Calgary, Alberta, Canada; ^3^Department of Surgery, Urology Section, University of Calgary, 1403 29 Street NW, T2N 2T9, Calgary, Alberta, Canada; ^4^Southern Alberta Institute of Urology, Office 6635, 7007 14 Street SW, T2V 1P9, Calgary, Alberta, Canada; ^5^Cancer Surveillance and Reporting, Alberta Health Services, 1400-10123 99 Street Edmonton, T5J 3H1, Calgary, AB, Canada; ^6^Department of Agricultural, Food and Nutritional Science and School of Public Health, University of Alberta, 116 Street & 85 Avenue, T6G 2R3, Edmonton, Alberta, Canada; ^7^Cancer Care Alberta, Alberta Health Services, 10030-107 Street NW, T5J 3E4, Edmonton, Alberta, Canada; ^8^Departments of Oncology, Community Health Sciences, and Mathematics and Statistics, University of Calgary, 2500 University Drive NW, T2N 1N4, Calgary, Alberta, Canada

## Abstract

Prostate cancer (PCa) stage at diagnosis is an important predictor of cancer prognosis. In Canada, over one-quarter of males are diagnosed with advanced-stage PCa. Studies have identified several factors associated with PCa stage at diagnosis; however, evidence from Canada is limited. This study aimed to examine associations between sociodemographic characteristics, health history, health practices, and psychosocial factors and PCa stage at diagnosis among males participating in Alberta's Tomorrow Project (ATP), a prospective cohort in Alberta, Canada. The study included males aged 35–69 years who developed PCa until January 2018. Factors associated with PCa stage at diagnosis were examined using partial proportional odds (PPO) ordinal regression models. A total of 410 males were diagnosed with PCa over the study period. A higher number of lifetime prostate-specific antigen tests were associated with earlier-stage PCa (OR 0.91, *p* = 0.02, 95% CI 0.83–0.99), while higher abdominal circumference (OR 1.02, *p* = 0.05, 95% CI 1.00–1.03), lower social support (OR 2.34, *p* < 0.01, 95% CI 1.31–4.17), and having children (OR 2.67, *p* < 0.01, 95% CI 1.38–5.16) were associated with later-stage disease. This study identified factors previously found in the literature as well as novel factors associated with PCa stage at diagnosis, which can help inform targets for cancer prevention programs to improve PCa prognosis.

## 1. Introduction

Prostate cancer (PCa) is the most common nonmelanoma cancer among males in Canada, accounting for 20% of all cancer cases and 10% of cancer-related deaths [1]. Cancer stage at diagnosis is a key indicator of cancer prognosis, with an earlier stage associated with greater treatment options and overall cancer survival [2]. In Alberta, Canada, the estimated 5-year relative survival rate among males diagnosed with earlier-stage PCa is 99% compared with only 48% among those with a late-stage diagnosis [3]. In 2016, nearly three-quarters (73%) of PCa cases in Alberta were diagnosed at an earlier stage (stage I or II) [3], aligning with national estimates [2]. However, over one-quarter of PCas were diagnosed at a late stage [3].

Prostate-specific antigen (PSA) testing was introduced to detect PCa in its early stages and reduce overall and cancer-related mortality [4]. However, evidence from several systematic reviews and meta-analyses of randomized controlled trials suggests that PSA testing may not effectively reduce all-cause or PCa-related mortality [5]. In addition, widespread screening using PSA testing has also led to overdiagnosis of nonaggressive PCa, leading to unnecessary, and sometimes harmful, treatment or testing [5]. As such, the Canadian Task Force on Preventive Health has recommended against population-based PSA screening among males of all ages [5].

Given current recommendations advising against PSA testing, identifying factors associated with advanced-stage PCa is essential to inform cancer prevention strategies. Previous studies have identified several sociodemographic, clinical, psychosocial, and health-related factors associated with PCa stage at diagnosis. For instance, family history of PCa, high comorbidity, African-American race, and older age have been associated with late-stage PCa diagnosis [6–8]. Males of lower socioeconomic positions (e.g., low income and lower educational level) and those with low social support and living in social isolation are also at a higher risk of advanced PCa at diagnosis [8–12]. In addition, while evidence pertaining to the influence of body composition and body size on PCa risk remains mixed, studies have found associations between higher fat mass, waist circumference, and body mass index (BMI) and advanced PCa at diagnosis [13, 14]. However, the majority of these studies were conducted in the United States, with little evidence from a Canadian context. Given cross-national differences in healthcare systems and health and social policies, identification of factors associated with late-stage PCa among males living in Canada can better inform Canadian cancer prevention and detection programs [15]. Therefore, the objective of this study was to examine associations between sociodemographic characteristics, health history, health practices, and psychosocial factors and PCa stage at diagnosis using data from Alberta's Tomorrow Project (ATP), a large prospective cohort in Alberta, Canada [16, 17].

## 2. Materials and Methods

### 2.1. Study Population

ATP is a prospective cohort of 55,000 healthy adults aged 35–69 years at enrollment living in Alberta [16, 18]. Individuals who were previously diagnosed with any cancer other than nonmelanoma skin cancer were excluded from enrolling. Participants completed questionnaires at baseline and subsequently every four years, with 31,211 participants completing the Health and Lifestyle Questionnaire (HLQ), Diet History Questionnaire, and Physical Activity Questionnaire between 2000 and 2008 [16, 18]. The HLQ domains include sociodemographic characteristics, health practices, psychosocial variables, and personal and family health history (Supplementary Materials (available here)) [16, 18].

**Figure 1:**
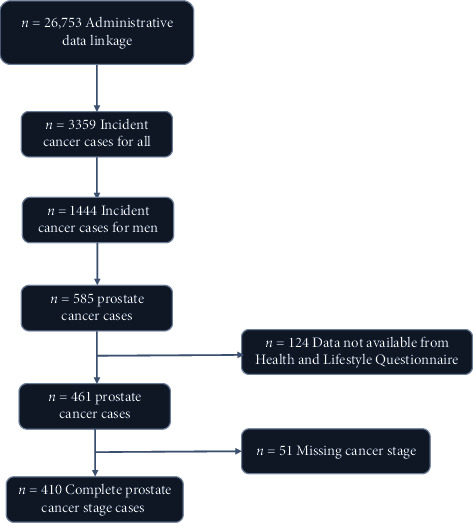
PRISMA diagram summarizing participants selection into the study.

A total of 1,867 males enrolled in ATP were diagnosed with invasive cancer (excluding nonmelanoma skin cancer) over the study period, including 585 males diagnosed with PCa. Inclusion criteria for the current study included male sex, completion of the HLQ (*n* = 461), and available data on stage at diagnosis (*n* = 410, *n* = 272 determined by the American Joint Committee on Cancer (AJCC) 7 ed., and *n* = 138 by AJCC 6 ed.) (Figure 1). Our final sample consisted of 410 males aged 35–69 years at baseline.

### 2.2. Outcome and Candidate Explanatory Variables

All PCa cases diagnosed from enrollment up until January 2018 from ATP were included in the analysis [16]. Dates of diagnosis and cancer stage were obtained for all participants who had consented to an annual linkage with the Alberta Cancer Registry, a provincial cancer registry mandated to collect diagnostic and death information for all Albertans diagnosed with cancer. PCa stage at diagnosis was defined by the TNM (tumour, node, and metastasis) staging system, with cancer stages ranging from stage I (small and contained) to stage IV (metastasized to other tissues) [2]. Due to the few numbers of males diagnosed in stages III and IV, they were combined into one group.

We identified candidate explanatory variables based on significance in the cited literature [8–14] or from novel variables collected in this study, including sociodemographic characteristics (e.g., annual household income), personal and family health history (e.g., self-rated health), psychosocial factors (e.g., social support), and health practices (e.g., cancer screening).

### 2.3. Statistical Analysis

We examined differences in candidate explanatory variables across PCa stages using analysis of variance for continuous variables and chi-square test for categorical variables. Binary variables were removed if one category had less than five responses, and categorical variables were retained with categories combined to increase subgroup size. Missing data were imputed using mean value replacement under a missing-at-random assumption for continuous variables. Missing categorical variables were replaced by the reference groups. Functional forms of the continuous variables and the influence of potential outliers were also assessed.

Since stage at diagnosis is an ordered categorial response variable (levels I–IV), proportional odds (PO) regression models were used rather than collapsing stage into two levels to fit a logistic regression model. This results in two cumulative logits not just one, making comparisons between stages I vs. II, III and IV, and I, II vs. III and IV for the association of explanatory variables possible. To overcome the constraint that the association of explanatory variables must be the same across every comparison or logit, partial proportional odds (PPO) models were used.

Variables were evaluated individually in PPO ordinal response models for association with PCa stage at diagnosis (*p* value <0.2), as well as meeting the PO assumption. All selected explanatory variables were then jointly evaluated in a multivariable PPO model with additional variables forced in based on relevance in the literature (e.g., family history of PCa) or on the conditional variable importance measure obtained from a random forest analysis (R package partykit, v1.2–5). Interactions with PCa screening variables were evaluated with the other explanatory variables in the final model due to their potential for effect modification.

Model checking was carried out using the available methods for the binary logistic setting in SAS (version 9.4) because many diagnostic tools were not implemented for the PPO model. Analyses were conducted in R (version 4.1.0, R Foundation for Statistical Computing, Vienna) and SAS version 9.4 (SAS Institute, Cary, NC). Statistical significance was set at *p* < 0.05.

## 3. Results

Among the 410 males included in this study, most were diagnosed with PCa at 60 years of age or older (72.7%), had a college or university education (73.0%), were married or common-law (86.3%), and were employed (70%) (Table 1). Most participants had a previous digital rectal exam (88.3%), while fewer had a previous PSA test (57.6%). Males diagnosed with late-stage PCa had fewer digital rectal exams and PSA tests, although they had a similar age at diagnosis distribution as males diagnosed at earlier stages. The majority (81.9%) of males did not have a family history of prostate cancer. Males tended to be overweight with a median BMI of 27.7 (quartile 1–quartile 3, 25.5–30.9) and were past or nonsmokers (88.0%). Over half (58.8%) reported having no stress at the time of survey completion and nearly three-quarters (71.5%) reported having 10 or fewer close friends or relatives who could provide social support.

The distribution of PCa stage at diagnosis was 14.6% at stage I, 69.0% at stage II, 10.5% at stage III, and 5.9% at stage IV. The median number of years from study entry to PCa diagnosis was 7 years (interquartile range: 4–9).

Table 2 summarizes the results of the final multivariable PPO model. PCa family history was not a significant predictor of stage at diagnosis (odds ratio (OR) 0.80, *p* = 0.42, 95% CI 0.46–1.38) but was forced into the model. Abdominal circumference was weakly associated with an increased risk of late-stage diagnosis (OR 1.02, *p* = 0.05, 95% CI 1.00–1.03). Participants with children had more than double the risk of late-stage PCa than those without children (OR 2.67, *p* < 0.01, 95% CI 1.38–5.16). Similarly, when comparing males diagnosed with stages II-IV PCa with those diagnosed with stage I, those with lower social support (having 10 or fewer close friends or relatives) had more than double the risk of late-stage PCa (OR 2.34, *p* < 0.01, 95% CI 1.31–4.17) but not for males diagnosed with stages III and IV versus stages I and II. The number of lifetime PSA tests was significantly protective against late-stage diagnosis (OR 0.91, *p* = 0.02, 95% CI 0.83–0.99), whereby for every additional lifetime PSA test, the odds of late-stage stage decreased by 9% in the PPO model with other variables held constant.

## 4. Discussion

This study identified both novel (i.e., having children) and previously identified factors (i.e., PSA testing, social support, and abdominal circumference) associated with PCa stage at diagnosis among males participating in a large prospective cohort study in Alberta, Canada. Among the 410 males who developed PCa, 16.4% (*n* = 67) were diagnosed with advanced-stage disease (stage III or IV), closely aligning with Canadian and Alberta statistics [2, 3]. The number of lifetime PSA tests was the only identified factor protective against late-stage diagnosis. In contrast, participants with 10 or fewer close friends or relatives, those with children, and those with a higher abdominal circumference had significantly higher odds of late-stage PCa.

This study found that the number of lifetime PSA tests was associated with earlier-stage PCa diagnoses. It is well established that PSA testing aids early detection of PCa [19–24], and similar to our results, findings from longitudinal randomized controlled trials have shown that repeated PSA testing is associated with a reduced risk of advanced-stage disease [24] and PCa mortality [25, 26]. For instance, findings from the Finnish Randomized Study of Screening for Prostate Cancer found that high-risk PCa incidence was highest amongst males screened only once, while two and three PSA screenings were associated with reduced risk of advanced PCa [24]. However, given the ongoing controversy regarding the risks and benefits of PSA screening, recommendations for repeated PSA testing must be further examined and balanced against the risk of PCa overdiagnosis and overtreatment. Additional PCa screening methods, implemented alone or in tandem with PSA testing, are being examined to reduce PCa overdiagnosis [27].

Our finding showed that lower levels of social support were associated with later-stage PCa diagnosis aligns with several previous studies [9, 12]. In a principle component analysis of neighborhood-level predictors of prostate cancer aggressiveness, Lynch et al. [12] identified that males living alone (OR 1.06, 95% CI 1.01, 1.11) and males older than 65 years living alone in a nonfamily household (OR 1.07, 95% CI 1.02, 1.13) were at a higher risk of aggressive PCa. Studies have also shown associations between marital status and a lower risk of metastatic or advanced PCa [28, 29]. The protective role of social support against advanced cancer diagnosis has been thought to be attributed to factors such as an individual's spouse, other family members, or friends encouraging cancer screening and regular physician follow-ups [28]. Marital status has also been associated with factors such as health practices (e.g., higher levels of physical activity) and higher household income, which may be protective against cancer development and progression [28–30].

Associations between having children and late-stage PCa diagnosis are not well documented. Several meta-analyses summarizing population-based case-control and cohort studies have reported moderate PCa risk reductions for males without children, ranging from 16% to nearly 20% [31–33]. A 2016 meta-analysis of 11 studies reported a significant reduction in PCa risk for childless males compared to males who had fathered at least one child; however, the heterogeneity across these studies was statistically significant (*p* < 0.001, *I*^2^ = 88.2%) suggesting further exploration of the association was required [34]. A previous meta-analysis summarizing 10 population-based case-control and cohort studies demonstrated no overall association between parental status and PCa risk [35].

This study found a weak association between abdominal circumference and increased risk of late-stage diagnosis. While evidence pertaining to the relationship between measures of excess body weight, such as BMI and waist circumference, and PCa risk remains mixed [36, 37], several studies have shown associations with advanced PCa [38]. The mechanisms underlying the role of excess body weight in advanced-stage cancer are thought to be due to higher levels of hormones and growth factors, such as insulin and leptin, and lower levels of testosterone, which may promote carcinogenesis [38]. Obesity is also associated with insulin resistance and low-grade chronic inflammation, further promoting cancer development [38].

This study addressed the limitations of previous research by expanding our understanding of the factors associated with advanced-stage PCa diagnosis among males living in Canada. We examined the associations between several important sociodemographic characteristics, psychosocial and health-related factors, and health practices and PCa stage at diagnosis using valid and reliable surveys and assessment tools in a large population-level cohort [39, 40]. Individual participant data were linked with multiple sources of administrative health data that have provincial coverage, thereby allowing the simultaneous examination of previously identified and novel factors. By using survey data collected prior to diagnosis, recall bias was minimized. The use of the PPO regression model in place of a simple logistic regression model was another strength, as we identified associations between social support and having children and PCa stage at diagnosis, which would have been otherwise missed.

Our study has several limitations including a higher proportion of ATP participants with postsecondary education and lower prevalence of daily smoking but a lower proportion of participants with a healthy BMI, compared to the Alberta weighted data from the Canadian Community Health Survey [16]. PSA values obtained over the life course were not available, and the reasons for males having or not having PSA tests were lacking. In addition, genetic variant data were not available that could provide additional insights. Finally, over 90% of ATP participants were of European descent, thus limiting the generalizability of these results.

## 5. Conclusion

Using high-quality population-level data, we examined a number of sociodemographic characteristics, health, and psychosocial factors associated with PCa stage at diagnosis in males. Abdominal circumference, having children, and low social support were significantly associated with late-stage PCa diagnosis, while the number of lifetime PSA tests was associated with earlier-stage diagnosis. Identifying factors associated with PCa stage at diagnosis can help inform targets for cancer prevention programs in Canada to improve PCa prognosis.

## Figures and Tables

**Table 1:** Characteristics of Alberta's Tomorrow Project's male participants diagnosed with prostate cancer, stratified by cancer stage at diagnosis (*n* = 410).

Characteristics	Stage I (*n* = 60) *n* (%)	Stage II (*n* = 283) *n* (%)	Stage III and IV (*n* = 67) *n* (%)	Total (*n* = 410) *n* (%)
Age at baseline (years)				
<50	11 (2.7)	42 (10.2)	15 (3.7)	68 (16.6)
50–59	28 (6.8)	119 (29.0)	27 (6.6)	174 (42.4)
60+	21 (5.1)	122 (29.8)	25 (6.1)	168 (41.0)
Age at diagnosis (years)				
<60	15 (3.7)	80 (19.6)	17 (4.1)	112 (27.3)
60–69	31 (7.6)	136 (33.2)	32 (7.8)	199 (48.5)
70+	14 (3.4)	67 (16.3)	18 (4.4)	99 (24.2)
Education				
High school diploma	14 (3.4)	79 (19.3)	18 (4.4)	111 (27.1)
College	30 (7.3)	133 (32.4)	32 (7.8)	195 (47.6)
University degree	16 (3.9)	71 (17.3)	17 (4.2)	104 (25.4)
Married or common-law				
Yes	49 (12.0)	247 (60.2)	58 (14.2)	354 (86.3)
No	11 (2.7)	36 (8.8)	−(2.2)	56 (13.7)
Employment				
Yes	44 (10.7)	198 (48.2)	45 (11.0)	287 (70.0)
No	16 (3.9)	85 (20.7)	22 (5.4)	123 (30.0)
Household income ($)				
<50 K	12 (2.9)	79 (19.3)	20 (4.5)	111 (27.1)
50–100 K	38 (9.3)	130 (31.8)	30 (7.3)	198 (48.8)
>100 K	10 (2.4)	73 (17.9)	17 (4.2)	100 (24.5)
Geography residence				
Rural	16 (3.9)	68 (16.6)	23 (5.6)	107 (26.1)
Urban	44 (10.7)	215 (52.4)	44 (10.7)	303 (73.9)
First degree PCa family history				
Yes	11 (2.7)	55 (13.4)	−(2.0)	74 (18.1)
No	49 (12.0)	228 (55.6)	59 (14.4)	336 (81.9)
Have ever had a digital rectal exam				
Yes	52 (12.7)	251 (61.2)	59 (14.4)	362 (88.3)
No	−(2.0)	32 (7.8)	−(2.0)	48 (11.7)
Have ever had a PSA test				
Yes	40 (9.8)	160 (39.0)	36 (8.8)	236 (57.6)
No	29 (4.9)	123 (30.0)	31 (7.6)	174 (42.4)
Have ever had a blood stool test				
Yes	29 (7.1)	100 (24.4)	26 (6.3)	155 (37.8)
No	31 (7.6)	183 (44.6)	41 (10.0)	255 (62.2)
Have ever had a sigmoidoscopy or colonoscopy				
Yes	18 (4.4)	70 (17.1)	15 (3.7)	103 (25.1)
No	42 (10.2)	213 (52.0)	52 (12.7)	307 (74.9)
Cancer grade^*∗∗*^				
1	29 (7.1)	16 (3.9)	0 (0)	45 (11.0)
2	30 (7.3)	126 (30.7)	14 (3.4)	170 (41.5)
3	0 (0)	138 (33.7)	50 (12.2)	188 (45.9)
Gleason score^*∗∗*^				
6	58 (14.2)	46 (11.2)	−(0.2)	105 (25.6)
7	0 (0)	93 (22.7)	25 (6.1)	118 (28.8)
8	0 (0)	15 (3.7)	−(1.7)	22 (5.4)
9	0 (0)	−(1.7)	11 (2.7)	18 (4.4)
10	0 (0)	0 (0)	−(0.5)	−(0.5)
NA	−(0.5)	122 (29.8)	21 (5.1)	145 (35.4)
Type of smoker				
Nonsmoker	29 (7.1)	113 (27.6)	25 (6.1)	167 (40.7)
Past smoker	26 (6.3)	134 (32.7)	34 (8.3)	194 (47.3)
Current smoker	−(1.2)	36 (8.8)	−(2.0)	49 (12.0)
Time spent in the sun 11 am–4 pm in June–August (hours)				
<1	48 (11.7)	205 (50.0)	51 (12.4)	304 (74.2)
≥1	12 (2.9)	78 (19.0)	16 (3.9)	106 (25.9)
Any stressful situations				
None	36 (8.8)	166 (40.5)	39 (9.5)	241 (58.8)
≥1	24 (5.8)	117 (28.5)	28 (6.8)	169 (41.2)
Number of close friends and relatives you have, social support^*∗*^				
≤10	34 (8.3)	213 (52.0)	46 (11.2)	293 (71.5)
>10	26 (6.3)	70 (17.1)	21 (5.1)	117 (28.5)
Have any children^*∗*^				
Yes	48 (11.7)	251 (61.2)	64 (15.6)	363 (88.5)
No	12 (2.9)	32 (7.8)	−(0.7)	47 (11.5)

	Median (Q1–Q3)	Median (Q1–Q3)	Median (Q1–Q3)	Median (Q1–Q3)

Age at baseline (years)	56 (50–63)	58 (53–63)	57 (50–63)	57 (52–63)
Distance to health centre by a vehicle (minutes)	9 (4–16)	8 (4–14)	7 (3–13)	8 (4–14)
Comorbidity index	0 (0-1)	0 (0-1)	0 (0-1)	0 (0-1)
Number of digital rectal exam in lifetime	4 (2–6)	4 (2–10)	3 (1–8)	4 (2–8)
Number of PSA blood tests in lifetime^*∗*^	1 (0–4)	1 (0–3)	0 (0–2)	1 (0–3)
Total recreational physical activity (MET hours/week)	23.3 (4.9–39.9)	24.3 (10.7–35.6)	24.9 (13.5–36.0)	24.9 (10.2–36.1)
Body mass index (kg/m^2^)	26.7 (24.6–30.0)	27.8 (25.6–30.9)	28.0 (26.3–30.9)	27.7 (25.5–30.9)
Abdominal circumference (cm)	96.5 (90.4–108.0)	101.8 (94.9–110.5)	101.6 (96.0–113.0)	101.6 (94.0–110.5)
Total dietary caloric intake (kcal/day)	1931 (1469–2239)	1931 (1522–2469)	1931 (1510–2267)	1931 (1505–2307)
Total alcohol intake (g/day)	6.5 (1.7–13.8)	7.2 (1.8–15.7)	6.7 (1.9–15.7)	7.1 (1.8–15.7)

Significant at ^*∗*^*p* < 0.05, ^*∗∗*^*p* < 0.01. “−” indicates number was <10 so cell entry suppressed. Comorbidity index: range (0–7). The range of the score of someone to take you to doctor if you needed it: 1: none of the time, 2: a little of the time, 3: some of the time, 4: most of the time, and 5: all of the time. Abdominal circumference was measured at one inch above the navel, even if this was not the usual waistline. PCa, prostate cancer; PSA, prostate-specific antigen.

**Table 2:** Predictors of cancer diagnosis at difference stages.

	Cancer stage^a^	OR (95% CI)	*p* value
PCa family history			
Yes vs. No		0.80 (0.46–1.38)	0.42
Abdominal circumference (cm)^b^		1.02 (1.00–1.03)	0.05
Number of close friends and relatives you have, social support			
>10		1.0	
≤10	IV and III vs. II and I	0.85 (0.48–1.50)	0.57
	IV and III and II vs. I	2.34 (1.31–4.17)	<0.01
Have any children^c^			
Yes vs. No		2.67 (1.38–5.16)	<0.01
Number of lifetime PSA tests		0.91 (0.83–0.99)	0.02

^a^If specified, refers to partial proportional odds; if no value specified, it is the proportional odds where coefficients are same for stages IV and III vs. II and I and IV and III and II vs. I. ^b^Abdominal circumference was measured at one inch above the navel, even if this was not the usual waistline. ^c^Step or grown children could be included in having any children response. OR, odds ratio; PCa, prostate cancer; PSA, prostate-specific antigen.

## Data Availability

Access to individual-level data is available in accordance with the Health Information Act of Alberta and Alberta's Tomorrow Project (ATP) Access Guidelines at https://myatpresearch.ca/DataAccess. Cancer registry data were obtained through linkage with Surveillance and Reporting, Cancer Research and Analytics, Cancer Care Alberta.

## Abbreviations

ATP: Alberta's Tomorrow Project
AJCC: American Joint Committee on Cancer
BMI: Body mass index
HLQ: Health and Lifestyle Questionnaire
OR: Odds ratio
PPO: Partial proportional odds
PCa: Prostate cancer
PSA: Prostate-specific antigen
TNM: Tumour, Node, and Metastasis.

## Supplementary Materials

Supplementary Materials The supplementary materials include the Health and Lifestyle Questionnaire.
