# Supplementary material for: Health-Related and Psychosocial Factors Associated with Prostate Cancer Stage at Diagnosis among Males Participating in Alberta's Tomorrow Project
Source: Prostate Cancer. 2023 Nov 10;2023:4426167. doi: 10.1155/2023/4426167 (PMC10656198; doi:10.1155/2023/4426167)
Supplement: Supplementary Materials — The supplementary materials include the Health and Lifestyle Questionnaire. [file 4426167.f1.pdf]

the tomorrow PROJECT
Albertans Studying the Connection Between Lifestyle and Cancer

Health and Lifestyle
Questionnaire

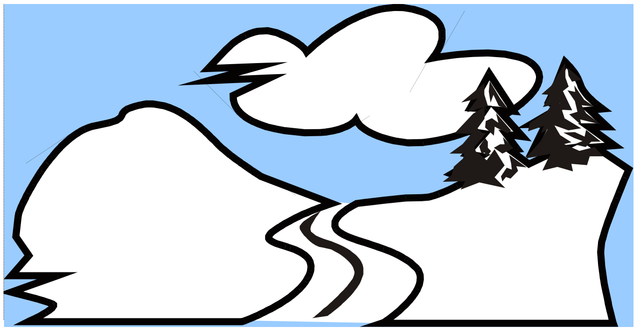

Variable List

2017-12-01

This box contains your unique study number and gender

HLQ_HR_2003 = Health Region 2003
999 = Missing
888 = NA
2222 = Not asked

Alberta Cancer Board

A research initiative of the Alberta Cancer Board

Office use only

○ L ○ C ○ V ○ QA

15916

Directions For Completing This Questionnaire

The Health and Lifestyle Questionnaire may take about 30 to 40 minutes to answer. Please follow the directions carefully. You will be asked to skip certain questions or whole sections that do not apply to you.

- ❖ We appreciate you completing the whole survey. However, if you prefer not to answer a question, write 'Decline' beside it.
- ❖ Use a pencil or a ballpoint pen, **not a felt pen**.

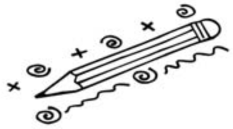

- ❖ Shade in the bubbles completely, like this: ●
- ❖ Write numbers in boxes like this: | 2 | 1 |
- ❖ If you make an error, put an X through the incorrect bubble like this:
- ❖ A tape measure is enclosed to take your body measurements on pages 28 and 29. Please report your measurements in feet, inches and pounds. The numbers will be changed to metric units at the study centre.
- ❖ Please leave the booklet stapled together - the pages will be separated at the study centre.

If you are not sure how to answer a question, please feel free to contact us:

Call our toll-free number in Canada: 1-877-919-9292.

Email us at: tomorrow@cancerboard.ab.ca

OR, for answers to commonly asked questions, check our website at www.thetomorrowproject.org

We are interested in your feedback about the questionnaire. Jot down your thoughts and suggestions in the space provided on the back cover of the blue booklet. We look forward to your input because it will help us to improve *The Tomorrow Project*® for other participants.

Sex: 1 = Male
2 = Female
3 = Transgender

15916

Section
A

Please **start** here
by answering these
questions about your personal health.

PHI 1 How would you rate your general health?

HLQ_PHI_1

1 ○ Excellent 3 ○ Good 5 ○ Poor

2 ○ Very good 4 ○ Fair

		Yes	No
PHI 2<br>HLQ_PHI_2	Has a doctor ever told you that you had cancer? (Do not include skin cancer unless it was melanoma.)	○<br>1	○<br>2

3 = Borderline/Insitu
99 = Presumed no

If yes, what type? ________________ ________________

HLQ_PRIOR_CANCER (derived - link to ACR)

Has a doctor ever told you that you had any of the following conditions?
(Shade yes or no for each condition.)

		Yes	No
PHI 3<br>HLQ_PHI_3	High blood pressure	1 ○	2 ○
PHI 4<br>HLQ_PHI_4	Angina (chest pains from a heart problem)	○	○
PHI 5<br>HLQ_PHI_5	High cholesterol in your blood	○	○
PHI 6<br>HLQ_PHI_6	Heart attack	○	○
PHI 7<br>HLQ_PHI_7	Stroke	○	○
PHI 8<br>HLQ_PHI_8	Emphysema	○	○
PHI 9<br>HLQ_PHI_9	Chronic bronchitis	○ 1	○ 2

		Yes	No
PHI 10<br>HLQ_PHI_10	Diabetes	1 ○	2 ○
PHI 11<br>HLQ_PHI_11	Polyps in your colon or rectum	○	○
PHI 12<br>HLQ_PHI_12	Ulcerative colitis	○	○
PHI 13<br>HLQ_PHI_13	Crohn's Disease	○	○
PHI 14<br>HLQ_PHI_14	Hepatitis	○	○
PHI 15<br>HLQ_PHI_15	Cirrhosis of your liver	○ 1	○ 2

99 = Presumed No

PHI 16 List any other long-term conditions that have lasted or are expected to last at least six months.

HLQ_CHRONIC_CONDITION

1. ________________ 2. ________________

3. ________________ 4. ________________

CHECKPOINT! Did you shade either yes or no for all the questions above?

15916

Section

B

This section is about your full blooded relatives' medical histories.
Do not include family members who are related to you by marriage or adoption. (Full-blooded sisters and brothers are those who had the same two biological parents as you.)

Note: If you are adopted, please include any family history that you know about, or choose "Don't Know" where appropriate.

FMH 1
HLQ_FMH_1_1

Have you ever had any full-blooded sisters who reached adulthood (age 21)?

1 ○ Yes → How many? ☐☐ Sisters
2 ○ No
3 ○ Don't know

HLQ_FMH_1_2

FMH 2
HLQ_FMH_2_1

Have you ever had any full-blooded brothers who reached adulthood (age 21)?

1 ○ Yes → How many? ☐☐ Brothers
2 ○ No
3 ○ Don't know

HLQ_FMH_2_2

FMH 3
HLQ_FMH_3_1

Have you ever had any daughters who reached adulthood (age 21)?
(If you currently only have daughters under 21, answer no.)

1 ○ Yes → How many? ☐☐ Daughters
2 ○ No
3 ○ Don't know

HLQ_FMH_3_2

FMH 4
HLQ_FMH_4_1

Have you ever had any sons who reached adulthood (age 21)?
(If you currently only have sons under 21, answer no.)

1 ○ Yes → How many? ☐☐ Sons
2 ○ No
3 ○ Don't know

HLQ_FMH_4_2

The next questions are about your natural (non-adoptive) mother and father.

FMH 5
HLQ_FMH_5

Is your natural mother still alive?

1 ○ Yes
2 ○ No → (Go to FMH 7)
3 ○ Don't know → (Go to FMH 8)

FMH 6
HLQ_FMH_6

How old is your mother now?

☐☐☐ Years → (Go to FMH 8)

15916

FMH 7 HLQ_FMH_7

How old was your mother when she died?

1 ○ Less than 40
2 ○ 40 - 49
3 ○ 50 - 59
4 ○ 60 - 69
5 ○ 70 - 79
6 ○ 80 - 89
7 ○ 90 - 99
8 ○ 100 years or older

FMH 8 HLQ_FMH_8

Is your natural father still alive?

1 ○ Yes
2 ○ No → (Go to FMH 10)
3 ○ Don't know → (Go to FMH 11)

FMH 9 HLQ_FMH_9

How old is your father now?

☐☐☐ Years → (Go to FMH 11)

FMH 10 HLQ_FMH_10

How old was your father when he died?

1 ○ Less than 40
2 ○ 40 - 49
3 ○ 50 - 59
4 ○ 60 - 69
5 ○ 70 - 79
6 ○ 80 - 89
7 ○ 90 - 99
8 ○ 100 years or older

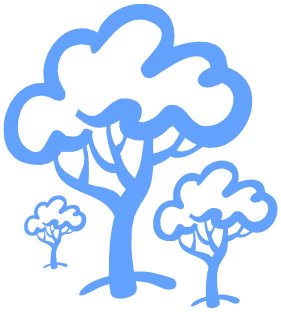

We would like to know if your **mother, father, full-blooded sisters, full-blooded brothers, daughters or sons** ever had any of the conditions listed on the next three pages.

If you are adopted, please include any information that you know about your biological family.

In future questionnaires we may ask for more detailed family histories about grandparents, aunts and uncles.

DIRECTIONS

- Enter the **age** each person was **first diagnosed**. (Your best guess)

OR

- **Shade the bubble** at the bottom of the page if, as far as you know, no one in your biological family has had the conditions listed.
- Leave the spaces blank if they do not apply to you.
- Look over the sample questions below then complete the charts on the next three pages.

Example of page 7

	Diabetes	Heart Attack	Stroke
Mother	62		
Father			87

62 is the age your mother was diagnosed with diabetes.

87 is the age your father first had a stroke.

Example of page 8

	Cancer of the Breast	Cancer of the Ovary	Cancer of the Rectum	Cancer of the Colon
Sister 1				
Sister 2	55			
Sister 3			65	
Sister 4				

55 is the age your second sister was diagnosed with breast cancer.

65 is the age your third sister was diagnosed with cancer of the rectum.

15916

FMH 11 Has anyone listed below been diagnosed with **diabetes, heart attack or stroke**?

- If YES, write the **age** the condition was **first diagnosed**.

OR

- If NO, **shade the bubble** at the bottom of the page. HLQ_FDR_CHRONIC_CONDITION

HLQ_FDR_CHRONIC_CONDITION_RELATIONSHIP

HLQ_FDR_CHRONIC_CONDITION_AGE

	Diabetes	Heart Attack	Stroke
**Mother**			
**Father**			
**Brother 1**			
**Brother 2**			
**Brother 3**			
**Brother 4**			
**Sister 1**			
**Sister 2**			
**Sister 3**			
**Sister 4**			
**Daughter 1**			
**Daughter 2**			
**Daughter 3**			
**Daughter 4**			
**Son 1**			
**Son 2**			
**Son 3**			
**Son 4**			

OR

○ To my knowledge, no one in my family listed above has had diabetes, a heart attack or a stroke.

15916

FMH 12 This chart is about cancer your full-blooded relatives may have had. Often cancer will start in one part of the body and then spread. **We are interested in where the cancer started.**

Has anyone been diagnosed with any of the following kinds of cancer?

- If YES, write the **age** the cancer was **first diagnosed**.

OR

- If NO, **shade the bubble** at the bottom of the page.

HLQ_FDR_CANCER_TYPE

HLQ_FDR_CANCER_AGE

HLQ_FDR_CANCER_RELATIONSHIP

Female Relatives

	Cancer of the Breast	Cancer of the Ovary	Cancer of the Rectum	Cancer of the Colon
**Mother**				
**Sister 1**				
**Sister 2**				
**Sister 3**				
**Sister 4**				
**Daughter 1**				
**Daughter 2**				
**Daughter 3**				
**Daughter 4**				

Male Relatives

	Cancer of the Breast	Cancer of the Prostate	Cancer of the Rectum	Cancer of the Colon
**Father**				
**Brother 1**				
**Brother 2**				
**Brother 3**				
**Brother 4**				
**Son 1**				
**Son 2**				
**Son 3**				
**Son 4**				

OR

○ To my knowledge, no one in my family listed above has had any of these cancers.

15916

Has anyone listed below been diagnosed with any other type of cancer?

- If YES, PRINT the **type of cancer** or **where it <u>started</u>** and the **age** it was first diagnosed.

OR

- If NO, **shade the bubble** at the bottom of the page.

Example

	Other Type of Cancer	Age
Father	Lung	7 9

HLQ_FDR_CANCER_RELATIONSHIP

	Other Type of Cancer	Age
Mother	HLQ_FDR_CANCER_TYPE	
Father		
Brother 1		
Brother 2		
Brother 3		
Brother 4		
Sister 1		
Sister 2		
Sister 3		
Sister 4		
Daughter 1		
Daughter 2		
Daughter 3		
Daughter 4		
Son 1		
Son 2		
Son 3		
Son 4		

HLQ_FDR_CANCER_AGE

OR

○ To my knowledge, no one in my family listed above has had any other type of cancer.

15916

Section

C

This section is about cancer screening tests.

SBB 1

HLQ_SBB_1

Have you ever had a digital rectal exam? (A digital rectal exam is when a doctor inserts a gloved finger into your rectum to check for cancer or other possible health problems.)

1 ○ Yes
2 ○ No ⟶ (Go to SBB 4)
3 ○ Don't know ⟶ (Go to SBB 4)

SBB 2

HLQ_SBB_2

When was the last time you had a digital rectal exam?

1 ○ Less than 6 months ago
2 ○ 6 months to less than 1 year ago
3 ○ 1 year to less than 2 years ago
4 ○ 2 years to less than 5 years ago
5 ○ 5 or more years ago

SBB 3

HLQ_SBB_3

About how many times in total have you had a digital rectal exam done? (Your best guess)

☐☐ Digital rectal exams

* * * * * *

SBB 4

HLQ_SBB_4

Have you ever had a blood stool test?

A blood stool test is collected at home, not at a doctor's office, to look for hidden blood in your stool. You have a bowel movement and use a small stick to smear a sample on a special card. You usually collect samples three days in a row.

1 ○ Yes
2 ○ No ⟶ (Go to SBB 8)
3 ○ Don't know ⟶ (Go to SBB 8)

SBB 5

HLQ_SBB_5

When was the last time you had a blood stool test done?

1 ○ Less than 6 months ago
2 ○ 6 months to less than 1 year ago
3 ○ 1 year to less than 2 years ago
4 ○ 2 years to less than 5 years ago
5 ○ 5 or more years ago

SBB 6

HLQ_SBB_6_

Why did you have the last blood stool test done? (Choose **ALL** that apply)

1 ○ Family history of colon or rectal cancer
2 ○ Part of regular checkup/routine screening
3 ○ Age
4 ○ Signs or symptoms of a possible problem
5 ○ Follow-up of previous problem
6 ○ Other (Please specify): ____________________

HLQ_SBB_6_OTHER

15916

SBB 7 HLQ_SBB_7

About how many times have you had a blood stool test done in your lifetime? (Your best guess)

[ ][ ] Blood stool tests

* * * * * *

SBB 8 HLQ_SBB_8

Have you ever had a sigmoidoscopy or colonoscopy done?

A sigmoidoscopy is an exam in which a doctor inserts a flexible tube into the rectum and lower part of the colon (lower bowel) to look for signs of cancer or other problems. The procedure may be done in a doctor's office or clinic and does not usually require sedation.

A colonoscopy is similar to a sigmoidoscopy but a longer tube is used to examine the entire colon. A colonoscopy is done in a clinic or hospital. Before the procedure is done, you are usually given medication through a needle in your arm to make you sleepy.

1 ○ Yes
2 ○ No → (**Men** go to Section D, page 12. **Women** go to Section F, page 14)
3 ○ Don't know → (**Men** go to Section D, page 12. **Women** go to Section F, page 14)

SBB 9 HLQ_SBB_9

When was the last time that you had a sigmoidoscopy or colonoscopy exam?

1 ○ Less than 6 months ago
2 ○ 6 months to less than 1 year ago
3 ○ 1 year to less than 2 years ago
4 ○ 2 years to less than 5 years ago
5 ○ 5 or more years ago

SBB 10 HLQ_SBB_10_

Why did you have the last sigmoidoscopy or colonoscopy test done? (Choose **ALL** that apply)

1 ○ Family history of colon or rectal cancer
2 ○ Part of regular checkup/routine screening
3 ○ Age
4 ○ Signs or symptoms of a possible problem
5 ○ Follow-up of previous problem
6 ○ Other (Please specify):

HLQ_SBB_10_OTHER ______________________

SBB 11

About how many times in total have you had either of these tests done in your lifetime?

HLQ_SBB_11_1 [ ][ ] Sigmoidoscopies

HLQ_SBB_11_2 [ ][ ] Colonoscopies

The cancer screening section is now complete.
MEN continue on the next page. WOMEN go to section F on page 14.

Section

D

This section is about **MALE** reproductive health. If you are **FEMALE**, go to Section F, page 14.

MRH 1 HLQ_MRH_1

Has a doctor ever told you that you have an enlarged prostate gland?

1 ○ Yes
2 ○ No
3 ○ Don't know

MRH 2 HLQ_MRH_2

Have you ever had surgery on your prostate gland?

1 ○ Yes
2 ○ No
3 ○ Don't know

MRH 3 HLQ_MRH_3

Have you ever had a vasectomy? (A sterilization procedure for men)

1 ○ Yes
2 ○ No
3 ○ Don't know

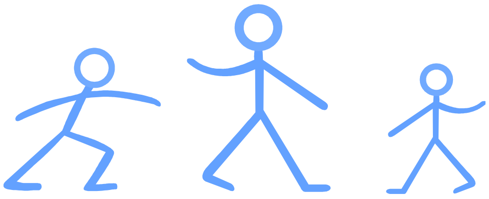

This section is about a **MALE** cancer screening test. If you are **FEMALE**, go to Section F, page 14.

SBM 1
HLQ_SBM_1

Have you ever had a PSA blood test? (This is a specific test ordered by a doctor to test men for prostate cancer.)

1 ○ Yes
2 ○ No → (Go to Section H, page 19)
3 ○ Don't know → (Go to Section H, page 19)

SBM 2
HLQ_SBM_2

When was the last time you had a PSA test?

1 ○ Less than 6 months ago
2 ○ 6 months to less than 1 year ago
3 ○ 1 year to less than 2 years ago
4 ○ 2 years to less than 5 years ago
5 ○ 5 or more years ago

SBM 3
HLQ_SBM_3_

Why did you have the last PSA test? (Choose **ALL** that apply)

1 ○ Family history of prostate cancer
2 ○ Part of regular checkup/routine screening
3 ○ Age
4 ○ Signs or symptoms of a possible problem
5 ○ Follow-up of previous problem
6 ○ Other (Please specify):

HLQ_SBM_3_OTHER

SBM 4
HLQ_SBM_4

About how many times in total have you had a PSA test in your lifetime? (Your best guess)

☐☐ PSA tests

The MALE cancer screening section is now complete.
MEN go to Section H, page 19.

15916

Section

F

This section is about **FEMALE** reproductive health. If you are **MALE**, go to Section H, page19.

FRH 1
HLQ_FRH_1

How old were you when you had your first menstrual period? (Your best guess)

1	2	3	4	5	6	7	8	9	10	11
○	○	○	○	○	○	○	○	○	○	○
9 or less	10	11	12	13	14	15	16	17	18 or more	Never had a period → (Go to FRH 3)

FRH 2
HLQ_FRH_2

How old were you when your periods first became regular? (Your best guess)

1	2	3	4	5	6	7	8	9	10	11
○	○	○	○	○	○	○	○	○	○	○
9 or less	10	11	12	13	14	15	16	17	18 or more	Never regular

FRH 3
HLQ_FRH_7_a

Have you ever been pregnant?

1 ○ Yes
2 ○ No → (Go to FRH 13)
3 ○ Don't Know → (Go to FRH 13)

FRH 4
HLQ_FRH_7_b

Are you currently pregnant?

1 ○ Yes → If yes, about how many weeks pregnant are you ? [|] Weeks
HLQ_WKS_PREG
2 ○ No
3 ○ Don't Know

FRH 5
HLQ_FRH_8

How many times have you been pregnant?

[|] Pregnancies

FRH 6
HLQ_FRH_9

Of your pregnancies, how many ended <u>before</u> 20 weeks?

[|] Pregnancies

FRH 7
HLQ_FRH_10

Of your pregnancies, how many lasted 20 weeks or more? (Include all pregnancies that ended in live births and still births)

[|] Pregnancies → (If you answered 0 pregnancies, go to FRH 13)

FRH 8
HLQ_FRH_11

How old were you when you completed your first pregnancy that lasted 20 weeks or more?

[|] Years

FRH 9
HLQ_FRH_12

Did you breast feed or nurse any children for at least one month?

1 ○ Yes
2 ○ No → (Go to FRH 13)

15916

FRH 10 HLQ_FRH_13

How many children did you breast feed for at least one month?

1 ○ 1
2 ○ 2
3 ○ 3
4 ○ 4
5 ○ 5
6 ○ 6
7 ○ 7
8 ○ 8 or more

FRH 11 HLQ_FRH_14

How old were you when you first breast fed a child for at least one month?

1 ○ Less than 20
2 ○ 20 - 24
3 ○ 25 - 29
4 ○ 30 - 34
5 ○ 35 - 39
6 ○ 40 - 44
7 ○ 45 or older

FRH 12 HLQ_FRH_15

Thinking about all the children you breast fed, how many months in total did you breast feed?

1 ○ 1 - 3 months
2 ○ 4 - 6 months
3 ○ 7 - 12 months
4 ○ 13 - 23 months
5 ○ 2 - 4 years
6 ○ More than 4 years

FRH 13 HLQ_FRH_16

Have you ever tried to become pregnant for more than one year without becoming pregnant?

1 ○ Yes
2 ○ No

FRH 14 HLQ_FRH_17

Between the time you had your first period, and your last period, did you ever go without having a period for at least one year? (Do not count times when you were pregnant or breast feeding.)

1 ○ Yes
2 ○ No
3 ○ Don't Know
4 ○ Never had a period

FRH 15 HLQ_FRH_18

Have you ever taken birth control pills for any reason? (Do not include birth control pills prescribed for menopause)

1 ○ Yes
2 ○ No → (Go to FRH 18)
3 ○ Don't Know → (Go to FRH 18)

FRH 16 HLQ_FRH_19

How old were you when you first started taking birth control pills?

1 ○ Less than 20
2 ○ 20 - 29
3 ○ 30 - 39
4 ○ 40 or older

FRH 17 HLQ_FRH_20

In total, how long have you taken birth control pills, other than for menopause? (Round to the nearest year.)

1 ○ Less than one month
2 ○ One month to 1 year
3 ○ 2 - 3 years
4 ○ 4 - 5 years
5 ○ 6 - 9 years
6 ○ 10 years or more

3 = 1 YEAR OR LESS

FRH 18 HLQ_FRH_3

Did you ever have an operation to have both of your ovaries removed?

1 ○ Yes
2 ○ No → (Go to FRH 20)
3 ○ Don't Know → (Go to FRH 20)

FRH 19 HLQ_FRH_4

At what age did you have both of your ovaries removed? (If you had 2 separate operations to remove your ovaries, please indicate your age at the time of your **last** surgery.)

[ ][ ] Years

FRH 20 HLQ_FRH_5

Did you ever have a hysterectomy? (An operation to have your uterus or womb removed)

1 ○ Yes
2 ○ No → (Go to FRH 22)
3 ○ Don't Know → (Go to FRH 22)

FRH 21 HLQ_FRH_6

At what age did you have your uterus (womb) removed?

[ ][ ] Years

FRH 22 HLQ_FRH_21_a

Have you had a natural menstrual period during the past 12 months? (Answer "No" if your bleeding was induced by hormone replacement therapy.)

1 ○ Yes → (Go to FRH 24)
2 ○ No
3 ○ Don't Know

FRH 23 HLQ_FRH_21_b

Did your menstrual periods stop occurring naturally? (Answer "No" if your periods stopped because of surgery, medication, pregnancy or breast feeding, or because you started hormone replacement therapy.)

1 ○ Yes → How old were you when you had your last "natural" period? [ ][ ] Years
2 ○ No
3 ○ Don't Know

HLQ_FRH_22_a
555 = COULD NOT BE DETERMINED

FRH 24 HLQ_FRH_23

Sometimes women take female hormones around the time of menopause. Have you **ever** used female hormones for menopause, e.g. tablets, pills, a patch or creams prescribed by a doctor?

1 ○ Yes
2 ○ No → (Go to Section G, page 17)
3 ○ Don't Know → (Go to Section G, page17)

FRH 25 HLQ_FRH_24

Are you **currently** using female hormones?

1 ○ Yes
2 ○ No

FRH 26 HLQ_FRH_25

In total, how long have you taken female hormones? (Round to the nearest year)

1 ○ Less than one month
2 ○ One month to 1 year
3 ○ 2 - 3 years
4 ○ 4 - 5 years
5 ○ 6 - 9 years
6 ○ 10 years or more

3 = 1 YEAR OR LESS

15916

Section

G

This section is about cancer screening tests for **FEMALES**. If you are **MALE**, go to Section H, page 19.

SBW 1
HLQ_SBW_1

Have you ever had a Pap smear test?

1 ○ Yes
2 ○ No → (Go to SBW 4)
3 ○ Don't know → (Go to SBW 4)

SBW 2
HLQ_SBW_2

When was the last time you had a Pap smear?

1 ○ Less than 6 months ago
2 ○ 6 months to less than 1 year ago
3 ○ 1 year to less than 3 years ago
4 ○ 3 years to less than 5 years ago
5 ○ 5 or more years ago

SBW 3
HLQ_SBW_3

About how many Pap smears have you had in your lifetime? (Your best guess)

☐☐ Pap smears

* * * * * *

SBW 4
HLQ_SBW_4

Have you ever had a mammogram (a breast x-ray)?

1 ○ Yes
2 ○ No → (Go to SBW 8)
3 ○ Don't know → (Go to SBW 8)

SBW 5
HLQ_SBW_5

When was the last time you had a mammogram?

1 ○ Less than 6 months ago
2 ○ 6 months to less than 1 year ago
3 ○ 1 year to less than 2 years ago
4 ○ 2 years to less than 3 years ago
5 ○ 3 years to less than 5 years ago
6 ○ 5 or more years ago

SBW 6
HLQ_SBW_6

How many mammograms in total have you had in your lifetime?

☐☐ Mammograms

15916

SBW 7
HLQ_SBW_7_

Why did you have your last mammogram? (Choose **ALL** that apply.)

1 ○ Family history of breast cancer
2 ○ Part of regular checkup/routine screening
3 ○ Age
4 ○ Previously detected lump
5 ○ On hormone replacement therapy
6 ○ Breast problem
7 ○ Other (Please specify): ____________
HLQ_SBW_7_OTHER

0 = NO 1 = YES

* * * * * *

SBW 8
HLQ_SBW_8

Other than a mammogram, have you ever had your breasts examined for lumps (tumors, cysts) by a doctor or health professional?

1 ○ Yes
2 ○ No → (Go to SBW 11)
3 ○ Don't know → (Go to SBW 11)

SBW 9
HLQ_SBW_9

When was the last time you had your breasts examined by a doctor or health professional?

1 ○ Less than 6 months ago
2 ○ 6 months to less than 1 year ago
3 ○ 1 year to less than 2 years ago
4 ○ 2 years to less than 5 years ago
5 ○ 5 or more years ago

SBW 10
HLQ_SBW_10

How many times in your lifetime have you had your breasts examined for lumps by a doctor or health professional? (Your best guess)

[ ][ ] Examinations

* * * * * *

SBW 11
HLQ_SBW_11

Have you ever examined your own breasts for lumps (tumors, cysts)?

1 ○ Yes
2 ○ No → (Go to Section H, page 19)
3 ○ Don't know → (Go to Section H, page 19)

SBW 12
HLQ_SBW_12

How often do you examine your breasts?

1 ○ At least once a month
2 ○ Once every 2 - 3 months
3 ○ Less often than every 2 - 3 months

The FEMALE cancer screening section is now complete.
Continue on the next page.

Section

H

The next set of questions is about your exposure to the sun in the past twelve months.

SUN 1
HLQ_SUN_1

In the past year, has any part of your body been sunburned? (A sunburn is any reddening or discomfort of your skin that lasts longer than 12 hours after exposure to the sun or other UV (ultraviolet) sources, such as tanning beds or sunlamps.)

1 ○ Yes
2 ○ No → (Go to SUN 4)

SUN 2
HLQ_SUN_2

In the past year, did any of your sunburns involve blistering?

1 ○ Yes
2 ○ No

SUN 3
HLQ_SUN_3

In the past year, did any of your sunburns involve pain or discomfort that lasted for more than 1 day?

1 ○ Yes
2 ○ No

SUN 4
HLQ_SUN_4

Would you say that the untanned skin color of your inner upper arm is:

1 ○ Light (white, fair, ruddy)
2 ○ Medium (olive, light brown, medium brown)
3 ○ Dark (dark brown, black)

SUN 5
HLQ_SUN_5

During this past June through August, on a typical day outdoors, approximately how much time did you spend in the sun between 11am and 4pm?

1 ○ Less than 30 minutes per day
2 ○ 30 minutes to less than 1 hour per day
3 ○ 1 to 2 hours per day
4 ○ Greater than 2 hours per day

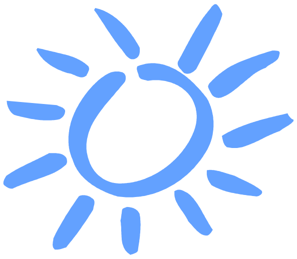

15916

Section

This section is about tobacco. The first questions are about **CIGARETTE SMOKING**. The term "cigarette" refers to cigarettes that are bought ready-made as well as those you roll yourself. Do not include cigars, cigarillos or pipes when you answer these first questions about cigarettes.

HLQ_SMK_STATUS (derived)

In this section, **read the directions and follow the arrows carefully**. There are different "paths" for nonsmokers, daily smokers, and occasional smokers.

SMK 1
HLQ_SMK_1

Have you smoked at least 100 cigarettes in your life? (About 4 - 5 packs)

1 ○ Yes → (Go to SMK 3)
2 ○ No
3 ○ Don't know

SMK 2
HLQ_SMK_2

Have you ever smoked a whole cigarette?

1 ○ Yes
2 ○ No → (Go to SMK 16a)
3 ○ Don't know → (Go to SMK 16a)

SMK 3
HLQ_SMK_3

At what age did you smoke your first whole cigarette?

[|] Years

SMK 4
HLQ_SMK_4

At the present time, do you smoke cigarettes daily, occasionally, or not at all?

1 ○ Daily (At least one cigarette every day for the past 30 days)
→ **If you smoke daily, continue with SMK 5**

2 ○ Occasionally (At least one cigarette in the past 30 days, but not every day)
→ **If you smoke occasionally, go to SMK 9 on page 21**

3 ○ Not at all (You did not smoke at all in the past 30 days)
→ **If you do not smoke at all, go to SMK 11 on page 21**

SMK 5
HLQ_SMK_5

At what age did you begin smoking cigarettes daily?

[|] Years

SMK 6
HLQ_SMK_6

How many cigarettes do you smoke each day now?

1 ○ 1 - 5 cigarettes
2 ○ 6 - 10 cigarettes
3 ○ 11 - 15 cigarettes
4 ○ 16 - 20 cigarettes
5 ○ 21 - 25 cigarettes
6 ○ 26+ cigarettes → If 26+, how many? [|] HLQ_SMK_6_1

15916

SMK 7
HLQ_SMK_7

For how many total years have you smoked daily?

[ ][ ] Years

SMK 8
HLQ_SMK_8

During the total years that you have smoked daily, about how many cigarettes per day have you usually smoked? (If your smoking pattern has changed over the years, make your best guess of the average number of cigarettes you have smoked per day.)

1 ○ 1 - 5 cigarettes
4 ○ 16 - 20 cigarettes
2 ○ 6 - 10 cigarettes
5 ○ 21 - 25 cigarettes
3 ○ 11 - 15 cigarettes
6 ○ 26+ cigarettes → If 26+, how many? [ ][ ] HLQ_SMK_8_1

→ **If you currently smoke daily, go to SMK 16a**

SMK 9
HLQ_SMK_9

On how many of the last 30 days did you smoke at least one cigarette?

1 ○ 1 - 5 days
3 ○ 11 - 20 days
2 ○ 6 - 10 days
4 ○ 21 - 29 days

SMK 10
HLQ_SMK_10

On the days that you smoked, how many cigarettes did you usually smoke?

1 ○ 1 - 5 cigarettes
4 ○ 16 - 20 cigarettes
2 ○ 6 - 10 cigarettes
5 ○ 21 - 25 cigarettes
3 ○ 11 - 15 cigarettes
6 ○ 26+ cigarettes

SMK 11
HLQ_SMK_11

Have you ever smoked cigarettes daily? (At least one cigarette a day for 30 days in a row)

1 ○ Yes
2 ○ No → (Go to SMK 16a)
3 ○ Don't know → (Go to SMK 16a)

SMK 12
HLQ_SMK_12

At what age did you begin to smoke daily?

[ ][ ] Years

SMK 13
HLQ_SMK_13

When you smoked daily, how many cigarettes did you usually smoke each day?

1 ○ 1 - 5 cigarettes
4 ○ 16 - 20 cigarettes
2 ○ 6 - 10 cigarettes
5 ○ 21 - 25 cigarettes
3 ○ 11 - 15 cigarettes
6 ○ 26+ cigarettes → If 26+, how many? [ ][ ] HLQ_SMK_13_1

SMK 14 For how many total years did you smoke daily?

HLQ_SMK_14

☐☐ Years

SMK 15 When did you stop smoking cigarettes daily?

HLQ_SMK_15

1 ○ Less than 1 year ago
2 ○ 1 to 2 years ago
3 ○ 3 to 5 years ago
4 ○ More than 5 years ago
5 ○ Don't know

→ Everyone answers the last questions.

SMK 16a Have you ever smoked cigarillos (e.g. Colts, Captain Black) at least once per week for 6 months or more?

HLQ_SMK_16_a

1 ○ Yes → For how many total years? ☐☐ How many per week? ☐☐☐ Cigarillos
2 ○ No
3 ○ Don't know

HLQ_SMK_16_a_1 HLQ_SMK_16_a_2

SMK 16b How often do you currently smoke cigarillos? 1 ○ Daily 2 ○ Occasionally 3 ○ Not at all

HLQ_SMK_16_b

SMK 16c Have you ever smoked cigars at least once per week for 6 months or more?

HLQ_SMK_16_c

1 ○ Yes → For how many total years? ☐☐ How many per week? ☐☐☐ Cigars
2 ○ No
3 ○ Don't know

HLQ_SMK_16_c_1 HLQ_SMK_16_c_2

SMK 16d How often do you currently smoke cigars? 1 ○ Daily 2 ○ Occasionally 3 ○ Not at all

HLQ_SMK_16d

SMK 16e Have you ever smoked a pipe at least once per week for 6 months or more?

HLQ_SMK_16_e

1 ○ Yes → For how many total years? ☐☐ How many per week? ☐☐☐ Pipes
2 ○ No
3 ○ Don't know

HLQ_SMK_16_e_1 HLQ_SMK_16_e_2

SMK 16f How often do you currently smoke a pipe? 1 ○ Daily 2 ○ Occasionally 3 ○ Not at all

HLQ_SMK_16_f

This section is complete.
If you are a NON SMOKER, continue with Section J, page 23.
If you CURRENTLY smoke cigarettes, cigars, cigarillos or a pipe DAILY or OCCASIONALLY, go to Section K, page 24.

15916

This Section is about second hand smoke and should be answered by people who **DO NOT SMOKE** at present.

If you **CURRENTLY** smoke cigarettes, cigars, cigarillos or a pipe either **DAILY** or **OCCASIONALLY (at least once in the last 30 days)**, please proceed to Section K, page 24.

SHS 1
HLQ_SHS_1

In the past year, were you exposed to second hand smoke on most days?

1 ○ Yes
2 ○ No

SHS 2
HLQ_SHS_2

In the past year, were you exposed to second hand smoke at home?

1 ○ Yes
2 ○ No

SHS 3
HLQ_SHS_3

In the past year, were you exposed to second hand smoke in a car or other private vehicle?

1 ○ Yes
2 ○ No

SHS 4
HLQ_SHS_4

In the past year, were you exposed to second hand smoke in public places? (bars, restaurants, shopping malls, arenas, bingo halls, bowling alleys)

1 ○ Yes
2 ○ No

SHS 5
HLQ_SHS_5

In the past year, were you exposed to second hand smoke when visiting friends or relatives?

1 ○ Yes
2 ○ No

SHS 6
HLQ_SHS_6

In the past year, were you exposed to second hand smoke in the work place?

1 ○ Yes
2 ○ No

Some studies have shown that stress can affect physical health. The following are stressful situations that sometimes come up in people's lives. As there are no right or wrong answers, the idea is to choose the answer **BEST** suited to your personal situation **AT THIS TIME**.

STR 1
HLQ_STR_1

You are trying to take on too many things at once.

1 ○ True

2 ○ False

STR 2
HLQ_STR_2

There is too much pressure on you to be like other people.

1 ○ True

2 ○ False

STR 3
HLQ_STR_3

Too much is expected of you by others.

1 ○ True

2 ○ False

STR 4
HLQ_STR_4

You don't have enough money to buy the things you need.

1 ○ True

2 ○ False

Please answer the next 3 questions if you are **married** or living **common-law** (living with a partner). If you are **single**, **widowed**, **separated** or **divorced**, go to STR 8.

Married or Common-law

STR 5
HLQ_STR_5

Your partner doesn't understand you.

1 ○ True

2 ○ False

STR 6
HLQ_STR_6

Your partner doesn't show you enough affection.

1 ○ True

2 ○ False

STR 7
HLQ_STR_7

Your partner is not committed enough to your relationship.

1 ○ True ⟶ (Go to STR 9)

2 ○ False ⟶ (Go to STR 9)

Single, Widowed, Separated or Divorced

STR 8
HLQ_STR_8

You find it difficult to find someone compatible with you.

1 ○ True

2 ○ False

The next 3 questions are about children

STR 9
HLQ_STR_9

Do you have any children? (Include grown children and step children.)
1 ○ Yes
2 ○ No ⟶ (Go to STR 12)

STR 10
HLQ_STR_10

One of your children seems very unhappy.
1 ○ True
2 ○ False

STR 11
HLQ_STR_11

The behaviour of one of your children is a source of serious concern to you.
1 ○ True
2 ○ False

Continue with these questions about your current situation

STR 12
HLQ_STR_12

Your work around the home is not appreciated.
1 ○ True
2 ○ False

STR 13
HLQ_STR_13

Your friends are a bad influence.
1 ○ True
2 ○ False

STR 14
HLQ_STR_14

You would like to move but can't.
1 ○ True
2 ○ False

STR 15
HLQ_STR_15

Your neighborhood or community is too noisy or polluted.
1 ○ True
2 ○ False

STR 16
HLQ_STR_16

You have a parent, a child or a partner who is in very bad health and may die.
1 ○ True
2 ○ False

STR 17
HLQ_STR_17

Someone in your family has an alcohol, drug or gambling problem.
1 ○ True
2 ○ False

STR 18
HLQ_STR_18

People are too critical of you or what you do.
1 ○ True
2 ○ False

Section L

Some studies have shown that the level of support we get from our friends and relatives can affect our physical health. Next are some questions about the support that is available to you.

SPT 1 About how many close friends and close relatives do you have (people you feel at ease with and can talk to about what is on your mind)?

Write in the number of close friends and close relatives Include your spouse and immediate family, if appropriate: [ ][ ] HLQ_SPT_1

How often is each of the following kinds of support available to you?	None Of The Time 1	A Little Of The Time 2	Some Of The Time 3	Most Of The Time 4	All Of The Time 5
SPT 2 HLQ_SPT_2 Someone to help you if you were confined to bed	○	○	○	○	○
SPT 3 HLQ_SPT_3 Someone you can count on to listen to you when you need to talk	○	○	○	○	○
SPT 4 HLQ_SPT_4 Someone to give you advice about a crisis	○	○	○	○	○
SPT 5 HLQ_SPT_5 Someone to take you to the doctor if you needed it	○	○	○	○	○
SPT 6 HLQ_SPT_6 Someone who shows you love and affection	○	○	○	○	○
SPT 7 HLQ_SPT_7 Someone to have a good time with	○	○	○	○	○
SPT 8 HLQ_SPT_8 Someone to give you information in order to help you understand a situation	○	○	○	○	○
SPT 9 HLQ_SPT_9 Someone to confide in and talk to about yourself or your problems	○	○	○	○	○
SPT 10 HLQ_SPT_10 Someone to hug	○	○	○	○	○
SPT 11 HLQ_SPT_11 Someone to get together with for relaxation	○	○	○	○	○
SPT 12 HLQ_SPT_12 Someone to prepare your meals if you were unable to do it yourself	○	○	○	○	○
SPT 13 HLQ_SPT_13 Someone whose advice you really want	○	○	○	○	○
SPT 14 HLQ_SPT_14 Someone to do things with to help you get your mind off things	○	○	○	○	○
SPT 15 HLQ_SPT_15 Someone to help you with daily chores if you were sick	○	○	○	○	○
SPT 16 HLQ_SPT_16 Someone to share your most private worries and fears with	○	○	○	○	○
SPT 17 HLQ_SPT_17 Someone to turn to for suggestions about how to deal with a personal problem	○	○	○	○	○
SPT 18 HLQ_SPT_18 Someone to do something enjoyable with	○	○	○	○	○
SPT 19 HLQ_SPT_19 Someone who understands your problems	○	○	○	○	○
SPT 20 HLQ_SPT_20 Someone to love you and make you feel wanted	○	○	○	○	○
	1	2	3	4	5

CHECKPOINT! Did you answer SPT 1 at the top of the page?

15916

Research suggests that people's feelings of spirituality may be related to their health.

For some people, being spiritual is similar to being religious; for other people, the ideas are different. Using a definition of spirituality that is most meaningful to you, please answer some questions about your spirituality.

SPI 1 HLQ_SPI_1

Do spirituality values or faith play an important role in your life?

1 ○ Yes

2 ○ No

SPI 2 HLQ_SPI_2

How religious or spiritual do you consider yourself to be?

1 ○ Not at all

2 ○ Not very

3 ○ Moderate

4 ○ Very

SPI 3 HLQ_SPI_3

People may practice or express their spirituality in many different ways, for example through prayer or meditation, or by attending services or gatherings. On average, during the past 12 months how often have you practiced your spirituality in some way?

1 ○ Daily or almost daily

2 ○ At least once a week

3 ○ At least once a month

4 ○ At least 3 - 4 times a year

5 ○ At least once a year

6 ○ Not at all

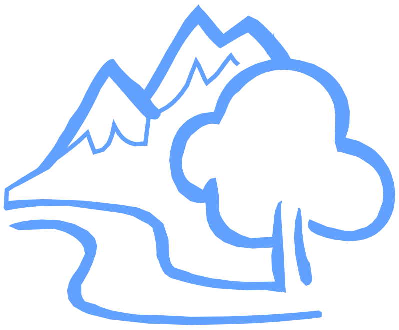

Section

N

In this part of the survey, we need you to take accurate measurements of your height, weight, abdomen, and buttocks.

Measurements should be made in a single session at least two hours after a meal, preferably with the help of another adult.
Weigh or measure yourself twice. Use the tape measure provided. The tape is divided in 1/8" sections.
Please record in feet, inches and pounds. The numbers will be converted to metric units at the study centre.

Height

1. Remove your shoes.
2. Stand straight with your back and heels against a wall.
3. Lay a book flat on top of your head and make a mark on the wall.
4. Measure twice. The two measurements should be within a quarter-inch (2/8 inch) of each other. If not, take a third measurement and record the closest two measurements.
5. Record your height in feet and inches.

5' 4": [5] Feet [4] Inches 6' 1½": [6] Feet [1.5] Inches

HLQ_HEIGHT (derived)

BOD 1	First Measurement:	[ ]	Feet	[ ]	Inches
BOD 2	Second Measurement:	[ ]	Feet	[ ]	Inches

Weight

1. Use a scale if possible to get your current weight. Adjust your scale to zero.
2. Remove your shoes and wear light clothing.
3. Weigh yourself twice. The two weights should be within one pound of each other. If not, weigh yourself a third time and record the closest two weights.
4. Record your weight in pounds.

HLQ_WEIGHT (derived)

BOD 3	First Measurement:	[ ]	Pounds
BOD 4	Second Measurement:	[ ]	Pounds

HLQ_BMI (derived)

15916

Abdomen and Buttocks

1. Take the next measurements either unclothed or in close fitting underwear.
2. Stand up straight in front of a mirror to position the measuring tape correctly.
3. Pull the tape measure so that it is snug and does not slide, but do not indent the skin.
4. Ensure that the tape is horizontal all the way around the body.
5. Measure twice. The two measurements should agree to within a quarter-inch of each other. If they do not, take a third measurement and record the closest two measurements.
6. Record the measurements in inches.

Abdomen

- Measure one inch above your navel or "belly button", EVEN IF THIS IS NOT YOUR USUAL WAISTLINE. See the diagrams below that show the correct measurement location.

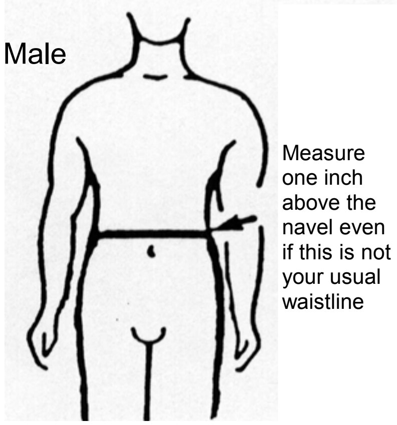

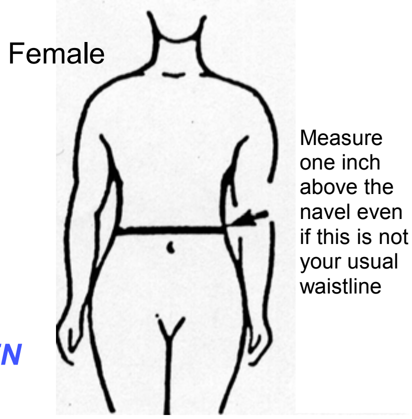

HLQ_ABDOMEN (derived)

BOD 5	First Measurement:	☐	Inches
BOD 6	Second Measurement:	☐	Inches

Buttocks

- Slide the tape measure up and down until you find the largest spot between your waist and thighs. See the diagrams below that show the correct measurement location.

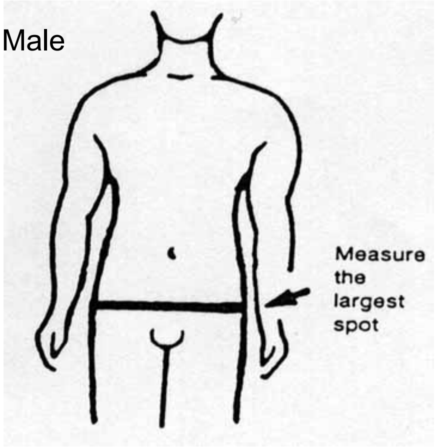

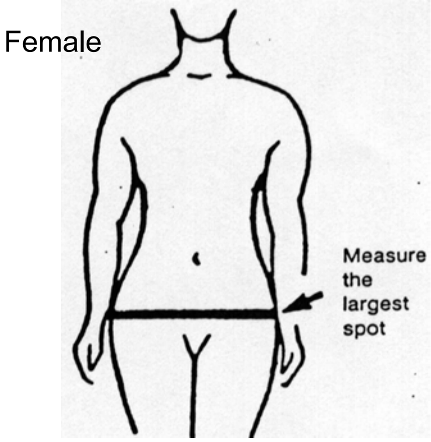

HLQ_HIP (derived)

BOD 7	First Measurement:	☐	Inches
BOD 8	Second Measurement:	☐	Inches

15916

Section O

We would like to ask you a few questions to describe yourself. Research has shown that there are connections between people's health and factors such as marital status, education, employment, income and ethnic background. **All information you provide will be kept completely confidential.**

DEM 1
HLQ_DEM_1

What is your current marital status? (Please choose the **ONE** status that best describes your current situation.)

1 ○ Married
2 ○ Divorced
3 ○ Not married, but living with someone
4 ○ Separated
5 ○ Widowed
6 ○ Single, never married

DEM 2
HLQ_DEM_2

What is the highest level of education you have finished? (Please choose **ONE**)

1 ○ Did not complete Grade 8
2 ○ Completed Grade 8, but not high school
3 ○ Completed high school
4 ○ Some technical school/college training completed
5 ○ Completed technical school/college training
6 ○ Some part of university degree completed
7 ○ Completed university degree
8 ○ Some part of post-graduate university degree completed
9 ○ Completed university post-graduate degree

DEM 3
HLQ_DEM_3

What is your current employment status? (Please choose the **ONE** that best describes your current situation. If you are self-employed, choose full-time or part-time as appropriate.)

1 ○ Working full-time (30 hours or more per week)
2 ○ Working part-time (Less than 30 hours per week)
3 ○ Not employed, but looking for work → (Go to DEM 6)
4 ○ Homemaker → (Go to DEM 6)
5 ○ Student → (Go to DEM 6)
6 ○ Retired → (Go to DEM 6)
7 ○ Other HLQ_DEM_3_OTHER ________ (Please Specify) → (Go to DEM 6)

DEM 4

If you currently work for pay, or are self employed, what type of work do you do in your job?

____

DEM 5

What is your job title?

____

15916

DEM 6
HLQ_DEM_7

The next question asks for your household income. We understand that this information is very private but the question is important for two reasons. Research has shown that there is a connection between income and health status. As well, the information helps to determine whether *The Tomorrow Project* includes a wide range of Albertans.

What was your approximate total **household** income before taxes last year?

(Please choose **ONE**)

1 ○ Less than $10,000
2 ○ $10,000 - $19,999
3 ○ $20,000 - $29,999
4 ○ $30,000 - $39,999
5 ○ $40,000 - $49,999
6 ○ $50,000 - $59,999
7 ○ $60,000 - $69,999
8 ○ $70,000 - $79,999
9 ○ $80,000 - $89,999
10 ○ $90,000 - $99,999
11 ○ $100,000 or more

DEM 7a

This final question asks about your ethnic origins, that is the ethnic or cultural groups to which your ancestors belonged. There is evidence that some ethnic groups are more likely to develop certain health problems and in addition, the information will help to determine if a wide range of Albertans have joined *The Tomorrow Project.*

What are your ancestral ethnic or cultural groups?
(Please choose **ALL** that apply)

○ British Isles (e.g. English, Irish, Scottish, Welsh)
HLQ_DEM_8_1

○ French (e.g. French, Acadian)
HLQ_DEM_8_2

○ Western European (e.g. Austrian, Belgian, Dutch, German, Swiss)
HLQ_DEM_8_3

○ Eastern European (e.g. Czech Republic, Hungarian, Polish, Romanian, Russian, Ukrainian)
HLQ_DEM_8_4

○ Northern European (e.g. Danish, Finnish, Icelandic, Norwegian, Swedish)
HLQ_DEM_8_5

○ Southern European (e.g. Albanian, Bulgarian, Croatian, Cypriot, Greek, Italian, Maltese, Portuguese, Serbian, Slovenian, Spanish, Yugoslav)
HLQ_DEM_8_6

○ East/Southeast Asian (e.g. Burmese, Cambodian, Chinese, Indonesian, Japanese, Korean, Vietnamese, Filipino)
HLQ_DEM_8_7

○ South Asian (e.g. Bangladeshi, Bengali, East Indian, Gujarati, Pakistani, Punjabi, Sinhalese, Sri Lankan, Tamil)
HLQ_DEM_8_8

○ West Asian (e.g. Afghan, Armenian, Iranian, Israeli, Kurdish, Turkish)
HLQ_DEM_8_9

○ Pacific Islands (e.g. Fijian, Hawaiian, Polynesian)
HLQ_DEM_8_10

○ Australian/New Zealander
HLQ_DEM_8_11

Choices continued on next page...

DEM 7 - continued from page 31

○ Arab/Middle Eastern (e.g. Egyptian, Iraqi, Lebanese, Maghrebi, Moroccan, Palestinian, Syrian)
HLQ_DEM_8_12

○ Latin/Central American (e.g. Costa Rican, Nicaraguan, Mexican, Salvadorian)
HLQ_DEM_8_13

○ South American (e.g. Argentinean, Bolivian, Brazilian, Chilean, Peruvian)
HLQ_DEM_8_14

○ North American (e.g. Canadian, American, Quebecois)
HLQ_DEM_8_15

○ Caribbean (e.g. Barbadian, Cuban, Guyanese, Haitian, Jamaican, Tobagonian, Trinidadian)
HLQ_DEM_8_16

○ African (e.g. Angolan, Black, Congolese, East African, Ethiopian, Kenyan, Nigerian, Somali, Ugandan)
HLQ_DEM_8_17

○ South African (e.g. Afrikaner)
HLQ_DEM_8_18

○ Aboriginal (e.g. North American Indian, Metis, Inuit)
HLQ_DEM_8_19

○ Other (Please specify) HLQ_DEM_8_OTHER ______________________
HLQ_DEM_8_20

○ Don't Know
HLQ_DEM_8_21

* * * * * *

FNL 1 What is your current age? [ ][ ] Years of age *HLQ_AGE_AT_SURVEY_RECEIPT*

FNL 2 Date survey completed __ __ / __ __ / __ __ __ __
M M D D Y Y Y Y

Thank you very much for answering the Health and Lifestyle Questionnaire.

Please return your questionnaire in the postage paid envelope at your earliest convenience.

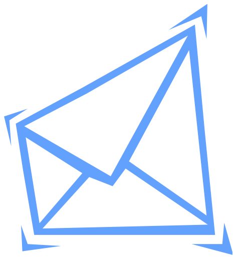

Comments? Record your comments or suggestions on the back of the blue Study Information Booklet.
